# Microbial contributions to coupled arsenic and sulfur cycling in the acid-sulfide hot spring Champagne Pool, New Zealand

**DOI:** 10.3389/fmicb.2014.00569

**Published:** 2014-11-04

**Authors:** Katrin Hug, William A. Maher, Matthew B. Stott, Frank Krikowa, Simon Foster, John W. Moreau

**Affiliations:** ^1^Geomicrobiology Laboratory, School of Earth Sciences, University of MelbourneMelbourne, VIC, Australia; ^2^Ecochemistry Laboratory, Institute for Applied Ecology, University of CanberraCanberra, ACT, Australia; ^3^Extremophiles Research Group, GNS ScienceWairakei, New Zealand

**Keywords:** arsenic speciation, thioarsenate, microbial diversity, hot springs, microbial arsenic resistance, sulfur cycling, Champagne Pool, New Zealand

## Abstract

Acid-sulfide hot springs are analogs of early Earth geothermal systems where microbial metal(loid) resistance likely first evolved. Arsenic is a metalloid enriched in the acid-sulfide hot spring Champagne Pool (Waiotapu, New Zealand). Arsenic speciation in Champagne Pool follows reaction paths not yet fully understood with respect to biotic contributions and coupling to biogeochemical sulfur cycling. Here we present quantitative arsenic speciation from Champagne Pool, finding arsenite dominant in the pool, rim and outflow channel (55–75% total arsenic), and dithio- and trithioarsenates ubiquitously present as 18–25% total arsenic. In the outflow channel, dimethylmonothioarsenate comprised ≤9% total arsenic, while on the outflow terrace thioarsenates were present at 55% total arsenic. We also quantified sulfide, thiosulfate, sulfate and elemental sulfur, finding sulfide and sulfate as major species in the pool and outflow terrace, respectively. Elemental sulfur concentration reached a maximum at the terrace. Phylogenetic analysis of 16S rRNA genes from metagenomic sequencing revealed the dominance of *Sulfurihydrogenibium* at all sites and an increased archaeal population at the rim and outflow channel. Several phylotypes were found closely related to known sulfur- and sulfide-oxidizers, as well as sulfur- and sulfate-reducers. Bioinformatic analysis revealed genes underpinning sulfur redox transformations, consistent with sulfur speciation data, and illustrating a microbial role in sulfur-dependent transformation of arsenite to thioarsenate. Metagenomic analysis also revealed genes encoding for arsenate reductase at all sites, reflecting the ubiquity of thioarsenate and a need for microbial arsenate resistance despite anoxic conditions. Absence of the arsenite oxidase gene, *aio*, at all sites suggests prioritization of arsenite detoxification over coupling to energy conservation. Finally, detection of methyl arsenic in the outflow channel, in conjunction with increased sequences from *Aquificaceae*, supports a role for methyltransferase in thermophilic arsenic resistance. Our study highlights microbial contributions to coupled arsenic and sulfur cycling at Champagne Pool, with implications for understanding the evolution of microbial arsenic resistance in sulfidic geothermal systems.

## Introduction

Active geothermal springs provide a modern analog for environments in which early life on Earth evolved metal(loid) resistance mechanisms (Stetter, 2006; Martin et al., 2008). In addition to high temperatures, high concentrations of dissolved toxic metal(loid)s present a strong selective pressure (Hirner et al., 1998) on extant hot spring microbial communities. Correspondingly, there is evidence to support the evolution of several microbial metal(loid) tolerance mechanisms in geothermal settings (Barkay et al., 2003; Jackson and Dugas, 2003; Maezato and Blum, 2012). In this regard, understanding the structure, diversity and functionality of modern hot spring microbial communities in the context of arsenic speciation may yield insights into the environmental conditions and constraints under which specific arsenic tolerance strategies evolved.

Arsenic is a metal(loid) that is toxic to microorganisms at elevated concentrations (Ballantyne and Moore, 1988) and can be present as several chemical species including the oxyanions arsenite (AsO^3−^_3_) and arsenate (AsO^3−^_4_) as well as arsenic thioanions. Arsenite has a high affinity to sulfhydryl groups in amino acids, thereby disrupting protein function (Oremland and Stolz, 2003). Arsenate is a phosphate analog, which displaces phosphate ions in enzyme reactions and therefore interferes with the cellular metabolism (Oremland and Stolz, 2003) or leads to mutagenic effects (Lièvremont et al., 2009). Previous work on arsenic speciation in geothermal environments reported the presence of primarily arsenite and arsenate contributing to the bulk arsenic speciation (Ballantyne and Moore, 1988; Yokoyama et al., 1993; Macur et al., 2004). However, improved sample preservation techniques, have revealed significant concentrations of thioarsenate species (Wilkin et al., 2003; Stauder et al., 2005; Planer-Friedrich et al., 2007; Wallschläger and Stadey, 2007), which can comprise more than 50% of the total dissolved arsenic in sulfidic waters (Wilkin et al., 2003). The presence of thioarsenates implies a potential dependence for arsenic speciation on sulfur redox cycling.

Sulfide, elemental sulfur, thiosulfate, and sulfate are common electron donors or acceptors for microorganisms under hydrothermal conditions (Amend and Shock, 2001; Kletzin et al., 2004; Gosh and Dam, 2009; Macur et al., 2013), and sulfide ions are highly reactive with arsenic (Sharma and Sohn, 2009). Thus, microbially-mediated sulfur cycling can exert a profound, although indirect, influence on arsenic speciation, specifically through controlling the relative abundance of thioarsenate species. In comparison to arsenite and arsenate, thioarsenates are considered to be less toxic for microorganisms, as the sulfur-arsenic bond leaves no free electron pair to bind with sulfhydryl-groups in amino acids (Stauder et al., 2005). Comparative genomic studies of the selenocysteine synthesis mechanism suggest thioarsenates may even be a microbial detoxification product in sulfur-rich environments (Couture et al., 2012). However, work by Planer-Friedrich et al. (2008) identified thioarsenate species as potentially toxic to microorganisms over longer exposure times.

Microbes employ a range of strategies to detoxify arsenic. The most ubiquitous arsenic resistance mechanism is the *ars* operon gene expression, which requires genes encoding for proteins that identify and transport arsenic (Paéz-Espino et al., 2009). The gene *arsC* expresses a reductase, which is able to convert arsenate into arsenite (Gladysheva et al., 1994), thereby providing resistance for arsenate. The gene *arsR* encodes for a transcriptional repressor, which controls the expression of the remaining *ars* operon genes *arsA, arsB, arsD, arsH*, and can only be activated by arsenite (Wu and Rosen, 1991). The gene *arsD* encodes for the metallochaperon ArsD that transfers arsenite to ArsA, which is an ATPase encoded by *arsA* and located at the cell membrane (Lin et al., 2007). The allosterically activated ArsA works as a catalytic subunit of ArsB, enhancing the activity of the membrane-located arsenite transporter that excludes arsenite from the cell (Rosen, 2002). In some cases the *ars* operon includes *arsH*, which encodes for an arsenite resistance enhancer ArsH, important at high arsenite concentrations (Branco et al., 2008). The gene *aio* (formerly known as *aox*, *aro* or *aso*) is a well-conserved arsenic resistance gene amongst several species that responds to degenerate primers (Quéméneur et al., 2008). It encodes for the arsenite oxidase Aio, which is responsible for the oxidation of arsenite into arsenate. Conversely, the highly diverse *arr* gene encodes for the respiratory arsenate reductase Arr in arsenate respiring microorganisms, which reduces arsenate into arsenite. A recent study by Richey et al. (2009) identifies a bidirectional enzyme Arr that is able to reduce arsenate as well as oxidize arsenite, implying an ancient origin. Despite the closer evolutionary relationship to Arr, Zargar et al. (2010, 2012) identify this gene as a new arsenite oxidase encoding gene referred to as *arxA*, because of its known function as an arsenite oxidase. The respiratory arsenate reductase and arsenite oxidase resistance mechanisms are both beneficial for microorganisms since they conserve energy for the cell (Paéz-Espino et al., 2009). Another arsenic resistance mechanism that microorganisms can apply involves methylation (Bentley and Chasteen, 2002). A study by Wallschläger and London (2008) detected mono- and dimethylated arsenic oxyanions in sulfidic groundwater, linking the presence of arsenic species with the activity of arsenic-methylating microorganisms. The relevance of methylated arsenic species in geothermal waters, and within the context of the evolution of arsenic resistance, is not yet well understood. To date, only a small number of thermophiles with arsenic-methylating activity have been identified (Qin et al., 2006; Takacs-Vesbach et al., 2013).

This study presents quantitative arsenic and sulfur speciation data, as well as cultivation-independent metagenomic analysis of microbial community structure and functional sulfur and arsenic gene inventories from Champagne Pool, an acid-sulfide hot spring at Waiotapu, New Zealand. The objectives of this work were (1) to determine potential microbial contributions to arsenic speciation in an extreme environment analogous to geothermal sites on the early Earth, (2) to characterize microbial diversity and richness at the 16S ribosomal RNA gene level across the hydrologic gradient of the pool and (3) to elucidate possible environmental constraints on the evolution of microbial arsenic resistance.

## Materials and methods

### Field site

The Taupo Volcanic Zone (TVZ) consists of a complex group of high temperature geothermal systems in the central North Island of New Zealand. One of the major geothermal fields in the TVZ is Waiotapu, which is characterized by a large number of springs with elevated arsenic concentrations (Hedenquist and Henley, 1985; Mountain et al., 2003). The largest feature at Waiotapu is Champagne Pool, ~65 m in diameter with an estimated volume of ~50,000 m^3^ (Hedenquist and Henley, 1985), and an arsenic concentration between 2.9 and 4.2 mg l^−1^ (this study). Champagne Pool is a geothermal surface feature and a source of high dissolved arsenite and sulfide concentrations (Childs et al., 2008). The inner rim of Champagne Pool is characterized by subaqueous orange amorphous As-S precipitate (Jones et al., 2001). The narrow outflow channel (~40 cm wide and 5 cm deep), in a subaerial sinter dam, drains the spring water out across a shallow siliceous sinter terrace. Convection in Champagne Pool stabilizes water temperatures to ~75°C within the pool itself, while on the surrounding silica terrace (“Artist's Palette”), the temperature decreases to ~45°C. Water-rock interactions beneath the pool that lead to silica dissolution and sulfide oxidation (Ellis and Mahon, 1964) provide sources of acidity to Champagne Pool waters. A high bicarbonate concentration, however, buffers the pH to ~5.5 (Hetzer et al., 2007). The precipitation of silica around the rim of Champagne Pool (Mountain et al., 2003) results in increased pH values toward Artist's Palette up to 6.9, favoring the dissolution of arsenic sulfide minerals that were precipitated inside the pool and washed out (Jones et al., 2001).

**Figure 1:**
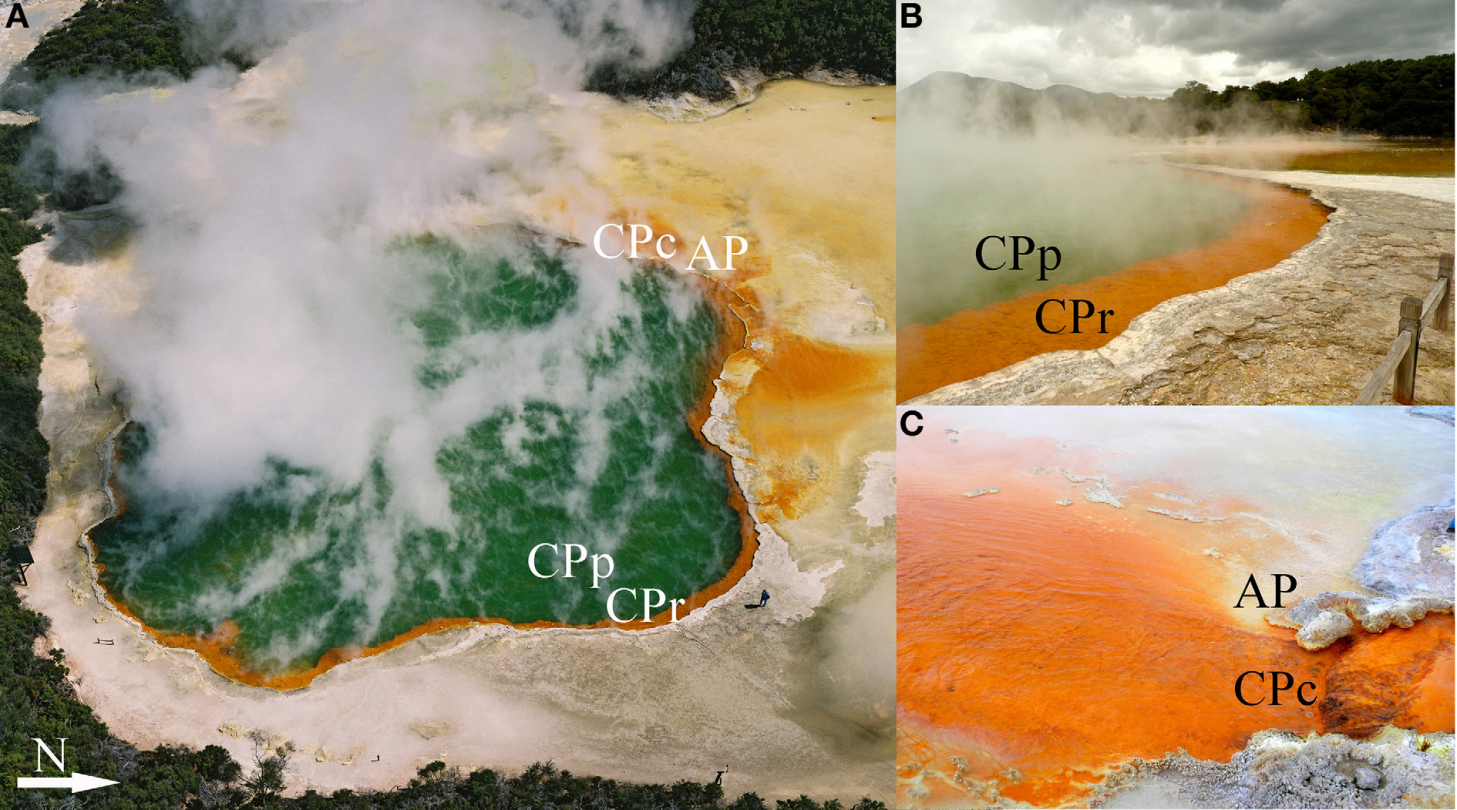
**Sampling sites (with abbreviations) at Champagne Pool, Waiotapu, New Zealand. (A)** Aerial view of Champagne Pool, photo credit: courtesy of GNS Science **(B)** CPp, central pool; CPr, rim of pool; **(C)** CPc, outflow channel (40 cm wide; 5 cm deep); AP, “Artist's Palette” terrace.

Four sampling sites at Champagne Pool were selected on the basis of distinctive physical and chemical characteristics. These sites were located along a natural hydrologic gradient from the inner pool (pool or “CPp”) through the inner rim (rim or “CPr”) and outflow channel (channel or “CPc”) on to an outer silica terrace (Artist's Palette or “AP”) (Figure 1). The AP samples were taken at a point immediately adjacent to CPc, where elemental sulfur precipitation was visible (Pope et al., 2004).

### Physical and chemical parameters

The pH, temperature, redox potential and DO (dissolved oxygen) saturation were measured *in situ* using a Professional Plus multimeter (YSI, USA). Water samples for dissolved organic carbon (DOC) were frozen at −20°C in the field and sent out for commercial analysis (Hills Laboratory, Hamilton, New Zealand), where the samples were filtered through a 0.45 μm nylon HPLC grade membrane filter and analyzed following the American Public Health Association APHA 5310-B Standard Method (Rice et al., 2012). Basic cations were measured using inductively coupled plasma atomic emission spectrometry (ICP-AES) (IRIS Intrepid II XDL, Thermo Corp). Chloride was measured using the potentiometric method following the American Public Health Association APHA 3500-Cl^−^ D Standard Method (Rice et al., 2012), and total bicarbonate was measured using the HCO^−^_3_ titration method following the ASTM Standards D513-82 (1988).

### Sampling and storage

Water samples for arsenic speciation analysis were stored in opaque 125 ml high-density polyethylene bottles (Nalgene, USA) that were washed with 1 M HCl and rinsed three times with sterile nano-pure water (Pall Corporation, USA) before a final rinse using the sample water immediately prior to sample collection. Water samples were collected via 50 ml sterile syringes (Terumo, USA), filtered through sterile 0.22 μm pore size Sterivex-GP polyethersulfone syringe filters (Merck Millipore, Germany) into the sample bottles, immediately flash frozen with liquid nitrogen, and placed into anoxic bags (BD Biosciences, USA). Frozen samples were transported on dry ice to the laboratory, where they were stored at −80°C until analysis. Immediately prior to arsenic speciation analysis, the samples were thawed under nitrogen in an anaerobic chamber to avoid oxidation.

Water samples for sulfur speciation and total sulfur analysis were collected via a portable peristaltic pump at 2 ml min^−1^ (Geopump Series II; Envco, Auckland, NZ). The sterile sample inlet tube made of silicon was placed directly into the sample site and the water was pumped directly from the springs into sterile polypropylene Falcon tubes (BD Biosciences, USA). The tubing was flushed thoroughly with spring water before taking samples. All samples, except those for elemental sulfur, were passed through a 0.45 μm pore size nylon filter (Merck Millipore, Germany) prior to collection in sterile Falcon tubes (BD Biosciences, USA). Additionally, 5% (w/v) zinc acetate (ZnAc) was added to the elemental sulfur samples in a 10:1 (v/v) ratio of sample:ZnAc to induce precipitation of (and thereby remove) zinc-sulfide from the sample. All sulfur samples, except the sulfide and total sulfur samples, were immediately frozen in liquid nitrogen and transported on dry ice to the laboratory, where they were stored at −80°C until analysis.

Sediment and water for DNA sequencing from each sample site except CPp were collected and stored in sterile polypropylene Falcon tubes (BD Biosciences, USA). Water from CPp was collected in a 5 l sterilized polypropylene vessel and immediately transported back to the laboratory with no temperature control. Approximately 500 ml volumes of CPp water were then filtered through a sterile 0.22 μm pore size cellulose membrane filter (Merck Millipore, Germany), collected and dried at room temperature on sterile petri dishes. The Falcon tubes and petri dishes with the sediment samples were stored at −80°C until further analysis.

### Preparation of standards

Stock solutions of arsenite, arsenate, methylarsonic acid (MA) and dimethylarsinic acid (DMA) were prepared as standards (1000 mg l^−1^) by dissolving sodium arsenite, sodium arsenate heptahydrate (AJAX Laboratory Chemicals), disodium monomethylarsenic and sodium dimethylarsenic (Sigma-Aldrich, Australia), respectively, in deionised water (Sartorius, Germany). Sodium monothioarsenate (Na_3_AsO_3_S^*^7H_2_O), sodium dithioarsenate (Na_3_AsO_2_S_2_^*^H_2_O), sodium trithioarsenate (Na_3_AsOS_3_^*^10H_2_O) and sodium tetrathioarsenate (Na_3_AsS_4_^*^8H_2_O) were synthesized in the lab using published protocols (Schwedt and Rieckhoff, 1996). Monomethylmonothioarsenate (MTMA) was synthesized by adding a saturated sulfide solution (deionized water purged with H_2_S for 1 h) to the monomethylarsenate (MA) standard and reacted for 30 min. Dimethylmonothioarsenate (MTDMA) was synthesized using the protocol by Raml et al. (2006). All thioarsenate standards were stored under nitrogen at 4°C. For the thiosulfate standard, 0.05 g of sodium thiosulfate (Na_2_S_2_O_3_^*^5H_2_O) was dissolved in 50 ml deionized water (Sartorius, USA) to obtain a thiosulfate concentration of 1000 mg l^−1^. For the sulfate standard, 0.1 g of sodium sulfate (NaSO_4_^*^10H_2_O) was dissolved in 200 ml deionized water (Sartorius, USA) to obtain a sulfate concentration of 500 mg l^−1^.

### Total arsenic and arsenic speciation analysis

Samples were thawed in a glove box filled with nitrogen gas. N_2_-purged deionized water (Sartorius, USA) was used to dilute samples when necessary. Total arsenic concentrations in water samples were measured in triplicate by electrothermal atomic absorption spectroscopy with a Perkin Elmer AAnalyst 600 graphite furnace using a previously published protocol (Deaker and Maher, 1999) with optimum concentrations of Pd/Mg [0.15 μmol (Pd) + 0.4 μmol (Mg)].

Arsenic speciation was measured using high-performance liquid chromatography coupled with inductively coupled plasma mass spectrometry (HPLC-ICPMS). Arsenic oxyanions were measured using a PEEK PRP-X100 anion exchange column (250 mm × 4.6 mm, 10 μm) (Phenomenex, USA). The mobile phase consisted of 20 mM ammonium phosphate buffer at pH 5.6, a flow rate of 1.5 ml min^−1^, column temperature of 40°C and injection volume of 40 μl (Kirby et al., 2004). Arsenic thioanions were measured using a 4 mm IonPac AG16 Guard and AS16 Analytical Column (Dionex, Sunnyvale, CA, USA) eluted with a NaOH gradient (1–100 mM) at 25°C and using a flow rate of 1 ml min^−1^ (Maher et al., 2013).

### Total sulfur and sulfur speciation analysis

Samples for sulfide analysis were fixed in the field, using the methylene blue method following the American Public Health Association APHA 3500-S2-D Standard Method (Rice et al., 2012). A volume of 50 ml of filtered sample from each site was collected and 1 ml of 1% (w/v) ZnAc solution (1 g dissolved in 100 ml degassed water) was added following three drops of 20 mM N,N'-dimethyl-p-phenylenediamine sulfate solution (7.4094 mg dissolved in 1 ml of 7.2 mM HCl). After 3 min incubation, 1.6 ml of 30 mM FeCl_3_ solution (4.866 mg dissolved in 1 ml of 1.2 mM HCl) was added. After returning to the laboratory, sulfide samples were measured using an UV/VIS spectrophotometer (Lambda 35 UV-Vis Spectrometer, Perkin Elmer) at extinction of 665 nm and with a detection limit of 0.01 mg sulfide kg^−1^. In order to be able to measure sulfide, the samples were diluted to within the standards concentration range of 0.04–1.5 mg l^−1^. In the laboratory, total sulfur samples were bubbled with oxygen for 15 min to oxidize all dissolved sulfur species into sulfate. The total sulfur concentration was measured using inductively coupled plasma spectrometry atomic emission spectroscopy (ICP-AES, IRIS Intrepid II XDL, Thermo Corp) using the American Public Health Association APHA 3120-B Standard Method (Rice et al., 2012). Validation of the results was obtained by the use of a certified quality control sample obtained from the New Zealand Accreditation Institute (IANZ). The thiosulfate, sulfate and elemental sulfur samples were thawed under nitrogen before analysis. Sulfate and thiosulfate concentrations were measured using HPLC UV spectrometry at 256 nm under the same conditions as described for the arsenic speciation. Prior to the elemental sulfur analysis, the elemental sulfur was extracted from the sample by shaking 40 ml of the samples with 5 ml toluene for 16 h, which dissolves at least 50 mg l^−1^ sulfur. After shaking, the toluene was withdrawn with a rubber-free syringe and filtered with a solvent-tolerant 0.2 μm pore size filter into a sterile 50 ml Falcon tube (BD Biosciences, USA) that was sent for commercial analysis (Geoscience Department, Southern Cross University, NSW, Australia). This method only extracts elemental sulfur, with sulfate and sulfide remaining in the water. The elemental sulfur samples were run on a HPLC reversed-phase silica column (Acclaim 120, Dionex). Methanol (95%) was used as the mobile phase at a flow rate of 1.6 ml min^−1^.

### SEM imaging

Environmental Scanning Electron Microscope (ESEM) photomicrographs of the precipitates observed at and collected from sites CPr and CPc were obtained with a FEI Quanta Scanning Electron Microscope (Bio21 Institute, University of Melbourne, VIC, Australia). Prior to analysis, the samples were centrifuged at 10,000 rpm for 10 min and excess water was decanted. The samples were stored at −20°C until analysis. Thawed samples were attached to sample holders and transferred to the ESEM chamber for microscopy under 0.8 mbar pressure.

### DNA extraction and quantification

The PowerSoil® DNA Isolation Kit (Mo Bio Laboratories, Carlsbad, USA) was used to extract total genomic DNA (gDNA) from the microbial communities in the sediments according to manufacturer protocol. The extracted DNA was stored at −20°C before further use. A NanoDrop 1000 Spectrophotometer (Thermo Fisher Scientific, USA) was used for the DNA quality determination at a wavelength ratio of A260/A280.

### Genomic DNA preparation for metagenomic sequencing

The gDNA was quantified using the Qubit dsDNA BR (Molecular probes®) assay system following manufacturer's protocol. Samples of sufficient quality were processed using the Illumina's Nextera XT sample preparation kit to generate Clean Amplified Nextera Tagment Amplicons (CAN) following manufacturer's protocol. CAN-DNA concentrations were checked using the Qubit dsDNA High sensitivity kit, while DNA fragment sizes were validated and quantified using the Agilent 2100 Bioanalyzer and Agilent high sensitivity DNA kit. The dilution factors for each sample library to obtain correct concentrations for sequencing on the MiSeq Sequencer were as follows: For library size of 250 bp from bioanalyzer the conversion factor for ng μl^−1^ to nM is 1 ng μl^−1^ = 6 nM, for library size of 500 bp from bioanalyzer the conversion factor for ng μl^−1^ to nM is 1 ng μl^−1^ = 3 nM, and for library size of 1000–1500 bp from bioanalyzer the conversion factor for ng μl^−1^ to nM is 1 ng μl^−1^ = 1.5 nM. Samples were diluted using Qiagen's EB (elution buffer) instead of Tris-Cl 10 mM 0.1% Tween 20.

### Illumina Miseq sequencing

Samples were processed for sequencing using the Illumina MiSeq reagent kit v2 (500 cycle) following a modified manufacturer's protocol. The modifications included: 1% (v/v) spike-in ratio of PhiX, the denatured DNA was diluted to a final concentration of 12 pM with pre-chilled HT1 buffer and Qiagen's EB solution instead of Tris-Cl 10 mM 0.1% (v/v) Tween 20 was used to dilute sequencing libraries and phiX throughout the protocol. Metagenomic sequencing was performed using the Illumina MiSeq machine (Peter Doherty Institute for Infection and Immunity, University of Melbourne, Australia).

### Metagenomic analysis

Sequence analysis was performed using the rapid annotation subsystems technology for metagenomes (MG-RAST) bioinformatics package, which is publicly available through http://metagenomics.anl.gov. Preprocessing steps included the removal of artificial sequences generated by sequencing artifacts (Gomez-Alvarez et al., 2009), and filtering any reads from the library that mapped to the *Homo sapiens* genome using Bowtie (Langmead et al., 2009). Furthermore, sequences were trimmed to contain at most five bases below a Phred score of 15, which was considered to be the lowest quality score included as a high-quality base. The maximum allowed number of ambiguous base pairs per sequence read was set to five. The numbers of sequences obtained by Illumina MiSeq sequencing was 3,843,368 for CPp; 3,146,467 for CPr; 2,926,799 for CPc and 4,623,251 for AP. The number of sequences after MG-RAST quality filtering was 2,461,097 for CPp; 2,392,176 for CPr, 2,176,985 for CPc and 3,969,176 for AP. MG-RAST used the SEED microbial genome annotation platform to determine the protein encoding genes of a metagenome via BLASTX. Sets of sequences were compared by grouping sets of annotations into higher-level functional groups. For taxonomic analysis, 16S rRNA gene sequence data were compared to all accessory databases (e.g., GREENGENES, RDP-II, etc.) by using search criteria specific for each database. Comparative analysis tools integrated into the MG-RAST pipeline were used to build rarefaction curves from 16S rRNA gene sequences detected at the Champagne Pool sites in order to investigate species richness. Graphics were generated with the R graphic program (R Development Core Team, 2013). The Fisher's exact test of independence was applied to functional gene distributions with the purpose of identifying significant differences (*p* < 0.05) in gene proportions from one Champagne Pool site to another.

## Results

### Water chemistry

The four sample sites at Champagne Pool showed similar physical and chemical conditions (Table 1). At sites CPp, CPr, and CPc, pH ranged between 5.5 and 5.8; while at site AP, pH increased to 6.9. All sites exhibited Eh values of ~ −117 to −15 mV (relative to the standard hydrogen electrode). The stable temperature in Champagne Pool (75°C) decreased toward the rim of the pool to 68°C and at Artist's Palette to 45°C. The DO saturation increased toward the margin of the pool to 45% at CPc. Dissolved organic carbon (DOC) concentrations declined below the detection limit of 0.5 mg l^−1^ at CPc. Dissolved iron concentrations were under the detection limit of 0.08 mg l^−1^ at all sites, and magnesium and aluminum were detected at concentrations ≤0.061 mg l^−1^ and ≤ 0.24 mg l^−1^ respectively (Table S1). In Champagne Pool the silica (as silicon) concentration was measured at ~490 mg l^−1^ and the bicarbonate (HCO^−^_3_) concentration was 127 mg l^−1^ (Table S1).

**Table 1:** **Temperature, pH, dissolved oxygen (DO) saturation, redox potential (Eh) and dissolved organic carbon (DOC) concentration in Champagne Pool**.

**Site ID**	**CPp**	**CPr**	**CPc**	**AP**
Site description	Central Champagne Pool	Rim Champagne Pool	Channel Champagne Pool	Terrace “Artist's Palette”
Image	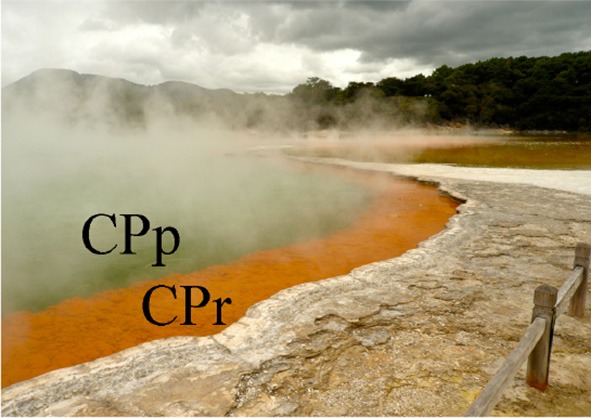		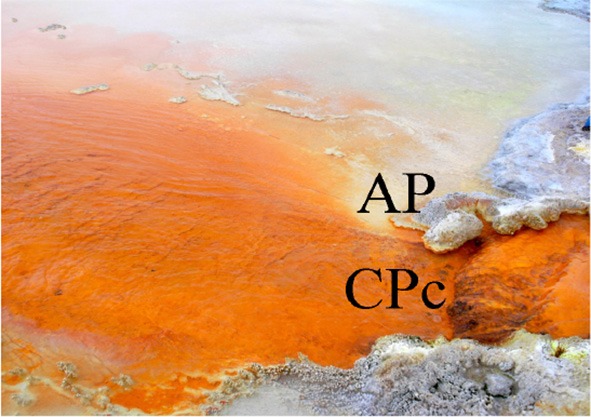	
Temperature (°C) (±0.2°C)	75	68	75	45
pH (±0.2 units)	5.5	5.5	5.8	6.9
Redox potential (mV) (±20 mV)	−117	−75	−74	−15
Dissolved oxygen (%) (±2%)	15	20	45	35
Dissolved organic carbon (mg l^−1^) (±5%)	2.2	4.1	<0.5	5.9

### Total arsenic concentrations and arsenic speciation

**Figure 2:**
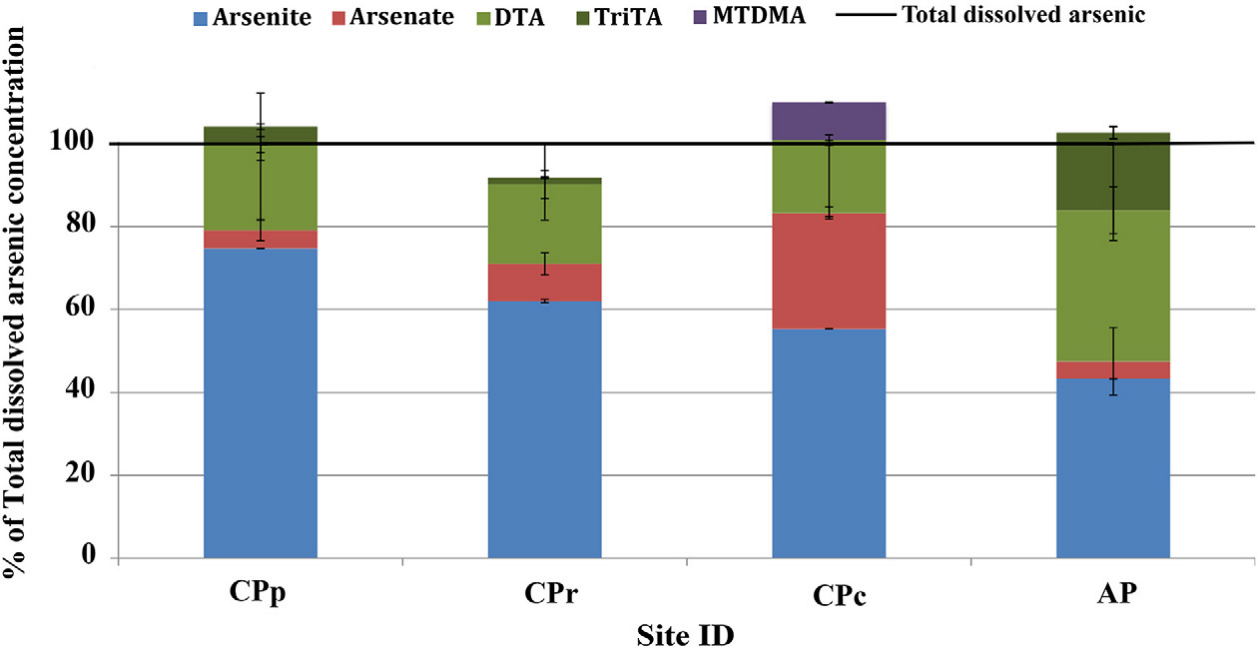
**Arsenic speciation as a percentage of total dissolved arsenic at Champagne Pool**. CPp, central pool; CPr, rim of pool; CPc, outflow channel; AP, “Artist's Palette” terrace. DTA, dithioarsenate; TriTA, trithioarsenate; MTDMA, dimethylmonothioarsenate.

Total dissolved arsenic concentrations of 3.0, 2.9, 3.6, and 4.2 mg l^−1^ were measured at sites CPp, CPr, CPc and AP, respectively (Table 2). The sum of arsenic species concentrations showed ≤10% difference from the total arsenic concentration at each Champagne Pool site (Table 2 and Figure 2). Changes in arsenic speciation occurred, however, along the sampling gradient at Champagne Pool (Table 2, Figure 2, and Figure S1). At sites CPp, CPr and CPc, arsenite was the major As species present, at between 55 and 75% of the total dissolved arsenic concentration; while at AP, thioarsenates were the primary detected species at 55% of the total dissolved arsenic concentration. CPp and CPr showed very similar proportions of arsenic species (Figure 2). A transition in As speciation occurred at CPc; however, where arsenate concentrations were observed to increase, the organic species dimethylmonothioarsenate (MTDMA) was first detected, and trithioarsenate (TriTA) was not detected (Figure 2). At Artist's Palette, arsenate concentrations decreased and no MTDMA was detected; however, the proportions of di- and trithioarsenate increased significantly (Figure 2). All Champagne Pool sites featured di- and trithioarsenate species; but noticeably, monothioarsenate was absent at all sites.

**Table 2:**
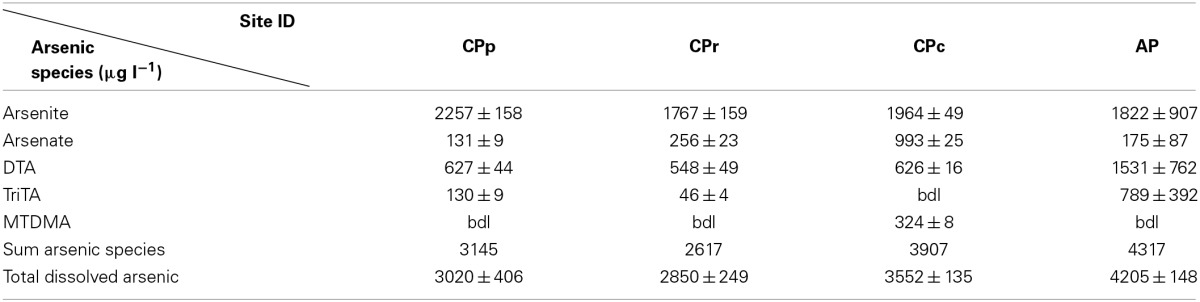
**Arsenic speciation and total dissolved arsenic concentrations in Champagne Pool**.

*CPp, central pool; CPr, rim of pool; CPc, outflow channel; AP, Artist's Palette. DTA, dithioarsenate; TriTA, trithioarsenate; MTDMA, dimethylmonothioarsenate. bdl, below detection limit (<0.01 μg l^−1^)*.

### Total sulfur concentrations and sulfur speciation

**Figure 3:**
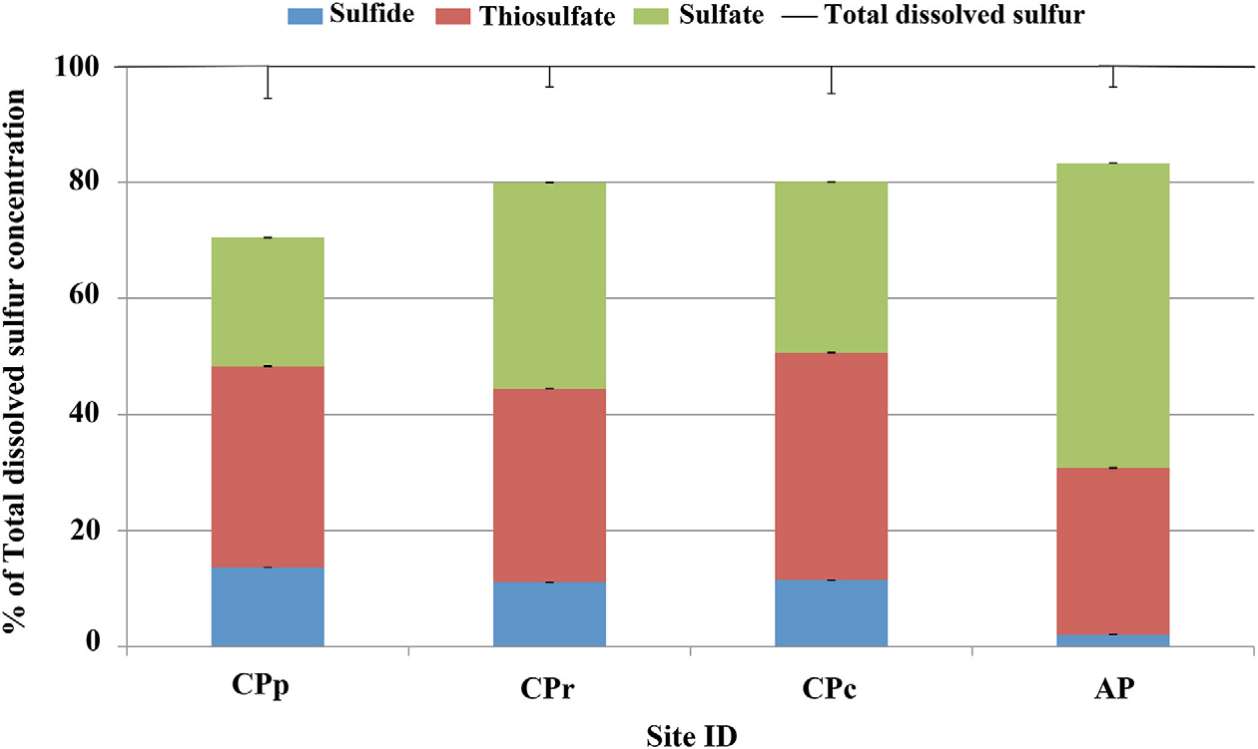
**Sulfur speciation as a percentage of total dissolved sulfur concentration at Champagne Pool**. CPp, central pool; CPr, rim of pool; CPc, outflow channel; AP, “Artist's Palette” terrace.

All sample sites contained total dissolved sulfur concentrations between 91 and 105 mg l^−1^ (Table 3). The sum of sulfur species showed recoveries of 70% (CPp), 80% (CPr and CPc), and 83% (AP) (Table 3 and Figure 3). A possible explanation for the observed difference between sulfur speciation and total dissolved sulfur concentrations could have been that some sulfur was present as sulfite (SO^2−^_3_), which was not quantified. Sulfur speciation changed across the sampling transect (Table 3 and Figure 3). The highest sulfide concentration was detected at CPp (12.6 mg l^−1^), before decreasing at the other sites. Thiosulfate showed stable concentrations throughout the sites (~30 mg l^−1^), whereas sulfate reached a maximum concentration at AP (55.1 mg l^−1^). Elemental sulfur, not included in the calculation of total (dissolved) sulfur, was detected at concentrations ≤1.7 mg l^−1^ at CPp, CPr and CPc, and at 34.8 mg l^−1^ at AP. Further SEM imaging of the precipitate, referred to as floc, at CPr and CPc, revealed a filamentous As-S phase and elemental sulfur colloids in a silica rich floc-sample (Figure S2).

**Table 3:**
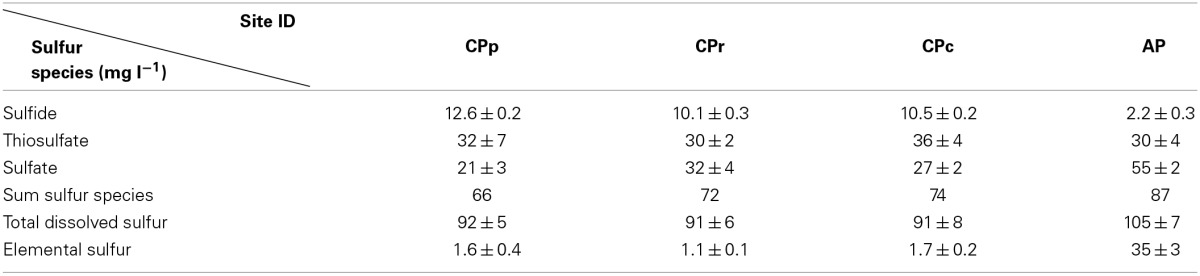
**Sulfur speciation and total dissolved sulfur concentrations in Champagne Pool**.

*CPp, central pool; CPr, rim of pool; CPc, outflow channel; AP, Artist's Palette*.

### Microbial 16S rRNA gene diversity

The richness of the microbial communities at the sample sites was characterized using bioinformatic analysis (Figure S3). The species abundance with increasing rarefaction sequence depths showed the highest species abundance at AP. The lowest species abundance was detected at CPc, with species abundances of CPp and CPr closer to CPp than to AP.

**Figure 4:**
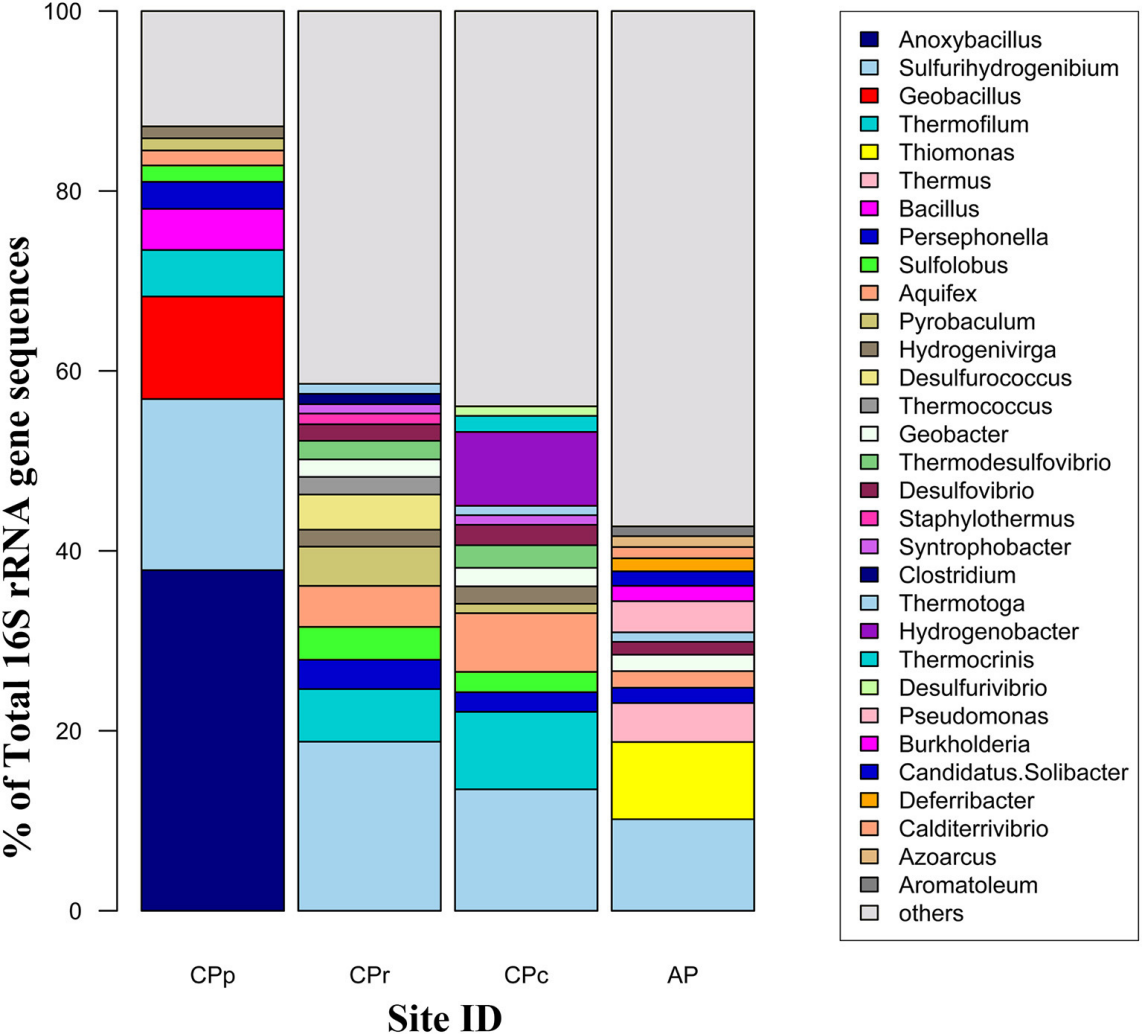
**Distribution of microbial 16S rRNA gene sequences (classified to genus level) at the four sample sites at Champagne Pool**. CPp, central pool; CPr, rim of pool; CPc, outflow channel; AP, “Artist's Palette” terrace. “Others” category in legend signifies genera present at <1% each.

The archaeal 16S rRNA gene diversity at all sites was analyzed at the genus level (Figure 4). At CPp, 12% of the sequences belonged to archaea, consisting almost exclusively of *Thermofilum, Sulfolobus and Pyrobaculum* (together 8%). At CPr and CPc, 16S rRNA gene sequences changed from ~12% (CPp) to ~21–28% (CPr, CPc) archaea with their members belonging mostly to genera *Sulfolobus*, *Thermofilum, Pyrobaculum, Desulfurococcus*, *Thermococcus*, and *Staphylothermus*. At AP, only 2% of the sequences belonged to archaea, with no dominant genus present.

The bacterial 16S rRNA gene diversity at all sites was analyzed at the genus level (Figure 4). Dominant sequences across all sites were most closely related to the genus *Sulfurihydrogenibium*. *Sulfurihydrogenibium*-related sequence abundance decreased continuously from 19% at CPp to 10% at AP. Additionally, bacterial genera *Anoxybacillus* and *Persephonella* comprised 38% and 3% of the total sequences at CPp, respectively. At CPr, members of the family *Sulfurihydrogenibium* were a dominant clade with 19% of the total sequences. Other major groups of bacteria from genera *Persephonella*, *Thermodesulfovibrio*, *Desulfovibrio*, *Thermotoga*, and *Syntrophobacter* represented 3.0%, 2.1%, 1.8%, 1.1%, and 1.0% of the total sequences, respectively. The dominant bacterial sequences at CPc were still related to *Sulfurihydrogenibium*, with an abundance of 13%. 16S rRNA gene sequences closely related to *Persephonella*, also belonging to the order *Aquificales*, comprised 2.2% of the total sequences. Additionally at CPc, bacteria belonging to the family *Aquificaceae* in genera *Aquifex, Hydrogenobacter, Hydrogenivirga*, and *Thermocrinis* represented 18% of the total sequences. Bacteria from genera *Thermodesulfovibrio* and *Desulfovibrio* comprised 3.6% of total sequences each at CPc. At AP, alongside *Sulfurihydrogenibium* (10% of total sequences), *Thiomonas* and *Thermus* were the most abundant bacterial genera in the community with 9% and 4% of total sequences.

### Relative abundance of functional arsenic resistance, sulfur metabolizing and hydrogenase encoding genes

Metagenomes analyzed with MG-RAST were searched for arsenic resistance, sulfur metabolism, and H_2_ respiration genes. The relative abundances of these genes are illustrated in Table 4.

**Table 4:** **Arsenic and sulfur genes found in the metagenome of Champagne Pool sites**.

	**CPp**	**CPr**	**CPc**	**AP**
% Arsenic resistance genes^a^	(0.07)	(0.09)	(0.1)	(0.1)
*arsB*	49 (0.04)	26 (0.02)	16 (0.02)	19 (0.02)
*acr3*	0.6 (0.0004)	15 (0.01)	16 (0.02)	31 (0.03)
*arsA*	14 (0.01)	34 (0.03)	44 (0.05)	15 (0.01)
*arsR*	11 (0.008)	8 (0.008)	4 (0.004)	8 (0.008)
*arsC*	26 (0.02)	17 (0.02)	20 (0.02)	25 (0.02)
*arsH*	0.1 (0.00007)	–	1 (0.001)
*arsD*	–	–	–	0.5 (0.0005)
% Respiration genes^b^	(2)	(3)	(4)	(3)
*arr subunit A*	0.002 (0.00004)	0.005 (0.0002)	–	0.03 (0.0009)
*arr subunit B*	–	–	–	0.06 (0.002)
*arr subunit C*	–	0.002 (0.00005)	–	–
Hydrogenase encoding genes	2 (0.05)	7 (0.2)	7 (0.3)	7 (0.2)
% Sulfur metabolizing genes^c^	(0.5)	(0.6)	(0.7)	(0.7)
Sulfur oxidation genes	8 (0.04)	8 (0.05)	11 (0.07)	24 (0.2)
Sulfur reduction genes	2 (0.009)	24 (0.1)	24 (0.2)	13 (0.1)
% Sulfur oxidation genes^d^	(0.04)	(0.05)	(0.07)	(0.2)
CcdA encoding gene	59 (0.02)	30 (0.01)	21 (0.02)	6.8 (0.01)
*sox* gene complex (without *soxCD*)	0.3 (0.0001)	5.7 (0.003)	63 (0.05)	47 (0.08)
*soxCD*	–	–	–	15 (0.03)
Sulfide dehydrogenase encoding gene	10 (0.004)	25 (0.01)	4 (0.003)	7 (0.01)
Sulfur oxygenase- reductase/Sulfite oxygenase encoding genes	30 (0.01)	37 (0.02)	12 (0.008)	8 (0.02)
% Sulfur reduction genes^e^	(0.009)	(0.1)	(0.2)	(0.1)
*dsrMKJOP* gene complex	72.8 (0.006)	62 (0.1)	63 (0.1)	59 (0.05)
*dsrC*	2 (0.0001)	7 (0.01)	6 (0.01)	5 (0.005)
*asrAB*	20 (0.002)	20 (0.03)	21 (0.04)	22 (0.02)

*arsB, arsenic efflux pump encoding gene; acr3, homolog to arsB; arsA, arsenical pump ATPase encoding gene; arsR, arsenic resistance repressor encoding gene; arsH, arsenite resistance enhancer encoding gene; arsD, metallochaperone transferase encoding gene; arr, respiratory arsenate reductase encoding gene; CcdA, protein for biogenesis of cytochrome c-type proteins; sox, sulfur oxidation genes; dsr, dissimilatory sulfite reductase encoding genes; asr, anaerobic sulfite reductase encoding gene. CPp, central pool; CPr, rim of pool; CPc, outflow channel; AP, Artist's Palette. Numbers in parentheses represent the % of the individual functional gene or gene complex on the total number of annotated genes in the microbial community at the given site*.

a *The percentage of the individual arsenic resistance gene on the overall arsenic resistance genes*.

b *The percentage of the arr arsenate reductase and hydrogenase genes on the overall respiration genes*.

c *The percentage of the sulfur oxidation and sulfur reduction genes on the overall sulfur metabolizing genes*.

d *The percentage of the individual sulfur oxidation gene or gene complex on the overall sulfur oxidation genes*.

e *The percentage of the individual sulfur reduction gene or gene complex on the overall sulfur reduction genes*.

Fisher's exact tests revealed significant differences in the proportion of *arsB* vs. *acr3* from CPp to all other sites (*p* = 0.00001), and CPr to AP (*p* = 0.02). Similarly, differences in the proportion of sulfur metabolism genes (sulfur oxidation to sulfur reduction or vice versa) were significant for CPp to CPr (*p* = 0.003), CPp to CPc (*p* = 0.01), CPr to AP (*p* = 0.002) and CPc to AP (*p* = 0.005).

## Discussion

### Geochemical influences on arsenic speciation

As a result of low or absent potential adsorbents such as the ox(yhydrox)ides of iron, magnesium and aluminum (Table S1), dissolved arsenic can accumulate in Champagne Pool waters to high concentrations (Table 2). Arsenic speciation in Champagne Pool is influenced by changes in the physiochemical conditions along the hydraulic gradient from the inner pool (CPp) to the Artist's Palette (AP) (Table 1). Analysis of arsenic and sulfur speciation in the context of Eh-pH stability fields (Lu and Zhu, 2011) predicts a predominance of arsenite at all sites, consistent with the relative percentage of arsenite measured at CPp, CPr, and CPc (Figure 2). HPLC-ICPMS analyses, however, showed significant thioarsenate concentrations at all sites, and furthermore revealed an organic arsenic species, dimethylmonothioarsenate, present at CPc. These observations were not consistent with the arsenic speciation predicted from redox potential and pH. Thus, both geochemical and microbial community analyses are required to understand arsenic transformations in Champagne Pool.

### Arsenic and sulfur cycling

**Figure 5:**
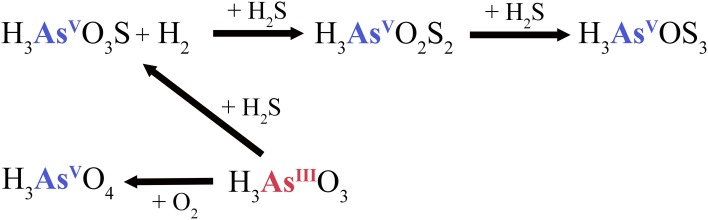
**Sulfide-dependent arsenic cycle at the pool (CPp) of Champagne Pool**. H_3_AsO_3_S, monothioarsenate; H_3_AsO_2_S_2_, dithioarsenate; H_3_AsOS_3_, trithioarsenate.

Hot springs with high sulfide or elemental sulfur concentrations contain higher proportions of thioarsenates, as sulfur has a higher affinity than oxygen for arsenic (Sharma and Sohn, 2009). All Champagne Pool sites contained significant amounts of thioarsenates, namely di-, and trithioarsenate (Figure 2). High sulfide concentrations at CPp (12.6 mg l^−1^) promote the transformation of arsenite into monothioarsenate and hydrogen (Figure 5). Planer-Friedrich et al. (2010) describe the arsenite transformation into thioarsenates via sulfide. No monothioarsenate was detected, however, as excess sulfide can further transform monothioarsenate into di- and trithioarsenate (Figure 5). The decrease in sulfide concentration from CPp to CPr is consistent with the observed increase in DO % saturation (Table 1) and loss of sulfide to precipitation (Figure S2). Lower trithioarsenate concentrations in CPr compared to CPp can be attributed to a slightly lower sulfide concentration (Table 3).

**Figure 6:**
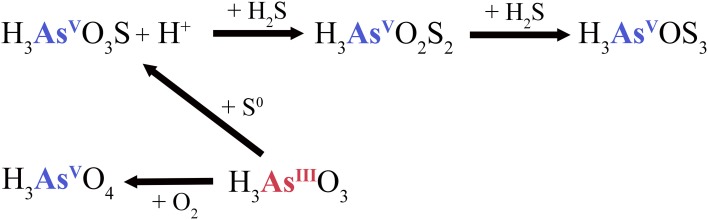
**Elemental sulfur-dependent arsenic cycle at the rim (CPr) and outflow channel (CPc) of Champagne Pool**. H_3_AsO_3_S, monothioarsenate; H_3_AsO_2_S_2_, dithioarsenate; H_3_AsOS_3_, trithioarsenate.

The arsenite transformation process in CPr and CPc may involve the activity of observed elemental sulfur (Figure S2), which transforms arsenite into monothioarsenate and H^+^ ions instead of H_2_ (Figure 6). Stauder et al. (2005) suggested the transformation of arsenite into thioarsenates via elemental sulfur. Further transformation of monothioarsenate into dithioarsenate would presumably be carried out via addition of sulfide, as abundant sulfide was also measured (Table 3). We estimate, based on the observations that similar sulfide concentrations were measured at CPr and CPc, but no trithioarsenate was detected at CPc, that the transformation from di- to trithioarsenate occured at a threshold of ~10 mg l^−1^ for dissolved sulfide (Table 3). Although relatively low elemental sulfur concentrations of 1.1 and 1.7 mg l^−1^ were detected in the water column of CPr and CPc, respectively, evidence for localized enrichment of S^0^ was found on filamentous precipitate detected at the rim and in the outflow channel (Figure S2). The shallow character of CPr and the increased DO saturation of 45% in CPc presumably facilitate the oxidation of sulfide into elemental sulfur (Xu et al., 1998, 2000).

**Figure 7:**
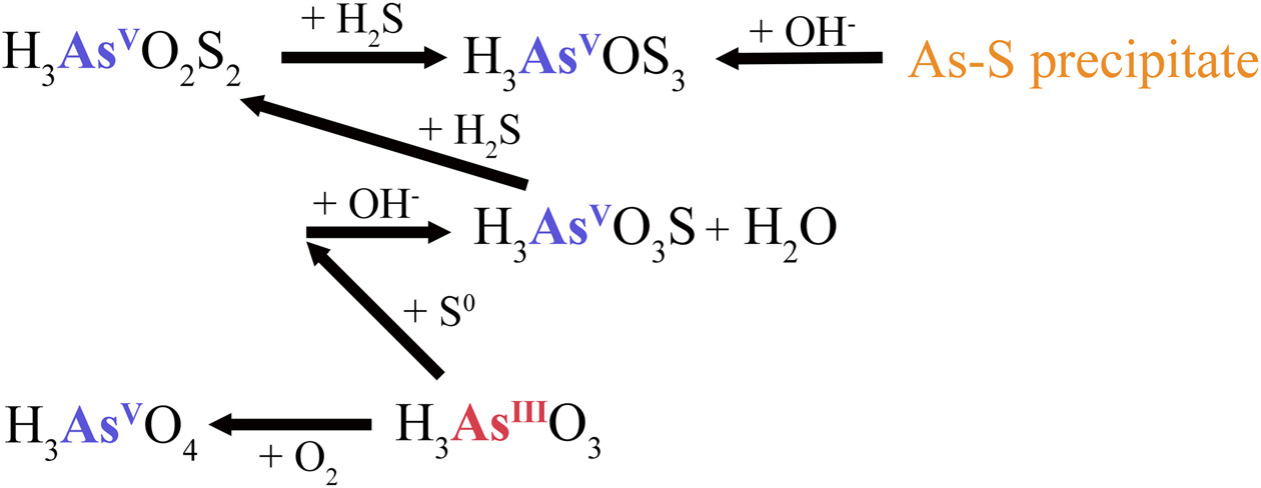
**Elemental sulfur-dependent arsenic cycle and dissolution of arsenic-sulfide precipitate at Artist's Palette (AP)**. H_3_AsO_3_S, monothioarsenate; H_3_AsO_2_S_2_, dithioarsenate; H_3_AsOS_3_, trithioarsenate.

Low sulfide concentrations of 2.2 mg l^−1^, and the increase of pH to 6.9 at AP, resulted in transformation via elemental sulfur to monothioarsenate and H_2_O (Figure 7). Furthermore, the pH increase at AP favors the dissolution of arsenic-sulfide precipitates detected at CPr and CPp (Figure S2), and conversion of As into di- and trithioarsenate (Figure 7), which explains their predominance adding to higher total dissolved arsenic concentrations (Table 2), despite low sulfide concentrations at AP. This interpretation is consistent with the observed higher solubility of arsenic-sulfide precipitates at higher pH (Webster, 1990; Eary, 1992). Elevated sulfur concentration in the water column of AP (Table 3) could be a result of elemental sulfur release during dissolution of the As-S precipitate (Figure S2).

### Microbial influences on the arsenic and sulfur cycle

As expected, microbial community analysis revealed increasing richness with decreasing temperatures and increasing pH (Figure S3). Community 16S rRNA gene sequences included a large group of sulfur-cycling microorganisms along the sampling transect at Champagne Pool, an observation which presents direct evidence to address previous hypotheses about the potential for indirect biological mediation of arsenic speciation via microbial sulfur cycling (Ulrich et al., 2013).

Despite reducing conditions at CPp (Table 1), thiosulfate and sulfate concentrations were elevated relative to sulfide (Table 3). The metagenome of the pool contained nearly 0.04% sulfur oxidation genes, in contrast with 0.009% sulfur reduction genes of the total annotated genes (Table 4), potentially explaining this observation. The combination of sulfide dehydrogenase and sulfur oxygenase-reductase encoding genes, detected as major sulfur oxidation genes at CPp (Table 4), suggests a two-step sulfide oxidation process to sulfite and thiosulfate, also producing sulfide. Thiosulfate could be further oxidized via oxygen at the surface of the pool. The annotation of sulfur oxidation genes detected at CPp (Table 4) to gene sequences from close relatives of detected 16S rRNA gene sequences (Figure 4) revealed a close relationship to genus *Sulfolobus*, which was detected in abundance at CPp (Figure 4). This sulfur-oxidizing genus (Brock et al., 1972) enhances the potential for production of thiosulfate and sulfate (Table 3). Other 16S rRNA gene sequences detected in the pool (CPp) were closely related to members of the order *Aquificales*, primary producers in high temperature ecosystems (Eder and Huber, 2002) and capable of oxidizing H_2_ or reduced sulfur species. The chemolithoautotrophic *Sufurihydrogenibium*, a genus of *Aquificales*, was a dominant phylotype detected along the gradient from CPp to AP (Figure 4). Although the relative abundance of *Sulfurihydrogenibium* decreased from CPp to AP, the genus remained a major component of the microbial communities at CPr, CPc, and AP (Figure 4). With only a few strains capable of oxidizing H_2_, the majority of this genus oxidizes S^0^ or S_2_O^2−^_3_ with O_2_ as the electron acceptor (Flores et al., 2008). *Sulfurihydrogenibium* as well as *Persephonella*, another member of the order *Aquificales*, which were the closest relatives of sequences detected at CPp (Figure 4), however, showed similarities in hydrogenase encoding genes (responsible for H_2_-oxidation) to those detected at this site (Table 4). The presence of sulfur reduction genes at CPp (Table 4), belonging to the *dsr* and *asr* gene complexes, is consistent with the thiosulfate- or elemental sulfur-reducing genus *Pyrobaculum* (Stetter et al., 1990) detected at the 16S rRNA gene level (Figure 4). This was further confirmed during the annotation of detected sulfur reduction genes (Table 4) with genes of close relatives from 16S rRNA gene sequences detected at CPp (Figure 4), which revealed a close relationship to representatives of genera *Pyrobaculum* and *Anoxybacillus* for the *dsr* and *asrAB* gene complexes. The resulting biogenic sulfide produced would then be available to transform arsenite to monothioarsenate (also yielding H_2_) (Figure 5).

At the rim of Champagne Pool, the increase in sulfur reduction genes (Table 4), belonging to the *dsr* and *asr* gene complexes, is consistent with the detection of close relatives from the genera *Thermofilum*, *Pyrobaculum*, *Desulforococcus*, *Staphylothermus*, *Thermococcus*, and *Thermotoga*, which have the potential to reduce thiosulfate or elemental sulfur to sulfide (Janssen and Morgan, 1992). Additionally, relatives of the observed genera *Thermodesulfovibrio*, *Desulfovibrio*, and *Syntrophobacter* can reduce sulfate to sulfide (Sekiguchi et al., 2008). The annotation of the detected sulfur reduction genes *dsr* and *asr* at CPr (Table 4) to genomic data from close relatives of 16S rRNA gene sequences detected at CPr (Figure 4) revealed a close relationship with genera *Pyrobaculum*, *Thermotoga*, *Desulfovibrio*, *Thermodesulfovibrio*, and *Syntrophobacter* for *dsr* and *asr*. Since the sulfide concentration at the rim did not increase significantly (Table 3), biogenic sulfide was probably rapidly reoxidized via microbial sulfur oxidation (e.g., *Sulfolobus*, detected at CPr; Figure 4). The annotation of major sulfur oxidation genes detected at CPr (Table 4), sulfide dehydrogenase and sulfur oxygenase-reductase encoding genes, with genes of close relatives of 16S rRNA genes detected at CPr (Figure 4) revealed a close relationship to members of the genus *Sulfolobus*. Alternatively, atmospheric oxygen could be responsible for the reoxidation of sulfide or biogenic sulfide could react with arsenite to form the ubiquitous orange arsenic-sulfide precipitates found around the rim of Champagne Pool (Figure S2). 16S rRNA gene sequences closely related to members of genera *Sulfurihydrogenibium* and *Persephonella* were dominant at CPr (Figure 4), and these genera most likely use H_2_ instead of reduced sulfur species as their electron donor, as supported by annotation of detected functional genes (Table 4) to genomic data of close relatives of 16S rRNA gene sequences detected at CPr (Figure 4).

At the rim and channel, the microbial community composition changed from ~12% (CPp) to ~21–28% archaea, with most archaeal phylotypes related to genera capable of heterotrophic sulfur-respiration, such as *Thermofilum, Pyrobaculum, Desulfurococcus*, *Thermococcus*, and *Staphylothermus* (Stetter et al., 1990) (Figure 4). The observed change suggests an increased bioavailability of dissolved organic carbon (DOC) in these locations. However, any DOC present in CPc samples was below the detection limit of 0.5 mg l^−1^, while CPp, CPr, and AP had measurable DOC concentrations (Table 1). Presumably, a relative increase in heterotrophs could have contributed to the disappearance of DOC at CPc. This interpretation leads to an interesting hypothesis that the first appearance of methylated arsenic species at Champagne Pool, in the channel site, is related to fundamental changes in microbial carbon utilization. The detection of 16S rRNA gene sequences closely related to *Thermodesulfovibrio* and *Desulfovibrio* (Figure 4) also supports the potential for bacterial sulfate reduction at this site, which is consistent with the sulfur reduction genes *dsr* and *asr* detected at CPr annotated to genes from the genera *Thermodesulfovibrio* and *Desulfovibrio*.

These changes in microbial community structure and sulfur speciation at CPr and CPc would likely result in a small decrease in H_2_ relative to background levels produced abiotically by geothermal reactions, i.e., if arsenite transformation to thioarsenate followed the reaction pathway with S^0^ instead of sulfide (Figure 6). Detection of a significant increase (greater than two standard deviations above the mean of sites CPp, CPr, and AP) in close relatives of *Aquificaceae* at CPc (Figure 4), however, with members *Aquifex, Hydrogenobacter, Hydrogenivirga, Thermocrinis* known for H_2_ oxidation (Eder and Huber, 2002), and detected hydrogenase encoding genes (Table 4) annotated to genes from these genera, confirm the persistence of H_2_ utilization as a primary energy source. Additionally, detected hydrogenase encoding genes from CPc (Table 4) could also be annotated to genes from genera *Sulfurihydrogenibium*, *Persephonella*, and *Thermofilum*, whose closely related sequences were detected at CPc (Figure 4).

The greater microbial species richness at AP (Figure S3), likely facilitated by decreased temperature of waters on the outflow terrace, establishes metabolic opportunities in terms of arsenic and sulfur cycling for a wider range of microorganisms. The increased proportion of sulfur oxidation vs. sulfur reduction genes detected at AP compared to CPr and CPc (Table 4), confirmed by Fisher's exact test, enables the oxidation of reduced sulfur compounds to sulfate. The annotation of the dominantly detected *sox* genes (including *soxCD*) (Table 4) to gene sequences from close relatives of detected 16S rRNA gene sequences at AP (Figure 4) revealed a close relationship to genera *Thermus*, *Thiomonas*, and *Sulfurihydrogenibium* (Figure 4 and Table 4). Members of the family *Thermus*, *Thiomonas*, and *Sulfurihydrogenibium* are known to oxidize elemental sulfur (Skirnisdottir et al., 2001; Dopson and Johnson, 2012) and the activity of these groups is consistent with the observed elevated SO^2−^_4_ concentrations at AP (Table 3). The significance of the change in sulfur oxidation vs. sulfur reduction gene proportion from CPp to CPr and CPc to AP, but not from CPp to AP, can be explained by a change in sulfur metabolism from CPp to CPr and CPc to AP (Table 4). At CPp, the main proportion of sulfur metabolizing genes belonged to sulfur oxidation genes, whereas at CPr, the dominant component of sulfur metabolizing genes belonged to sulfur reduction genes (Table 4). From CPc to AP the proportion of sulfur metabolizing genes changed again from sulfur reduction genes at CPr and CPc to sulfur oxidation genes at AP (Table 4). This trend is reflected by the sulfur species detected at the individual site (Table 3) and the microbial communities present (Figure 4).

**Figure 8:**
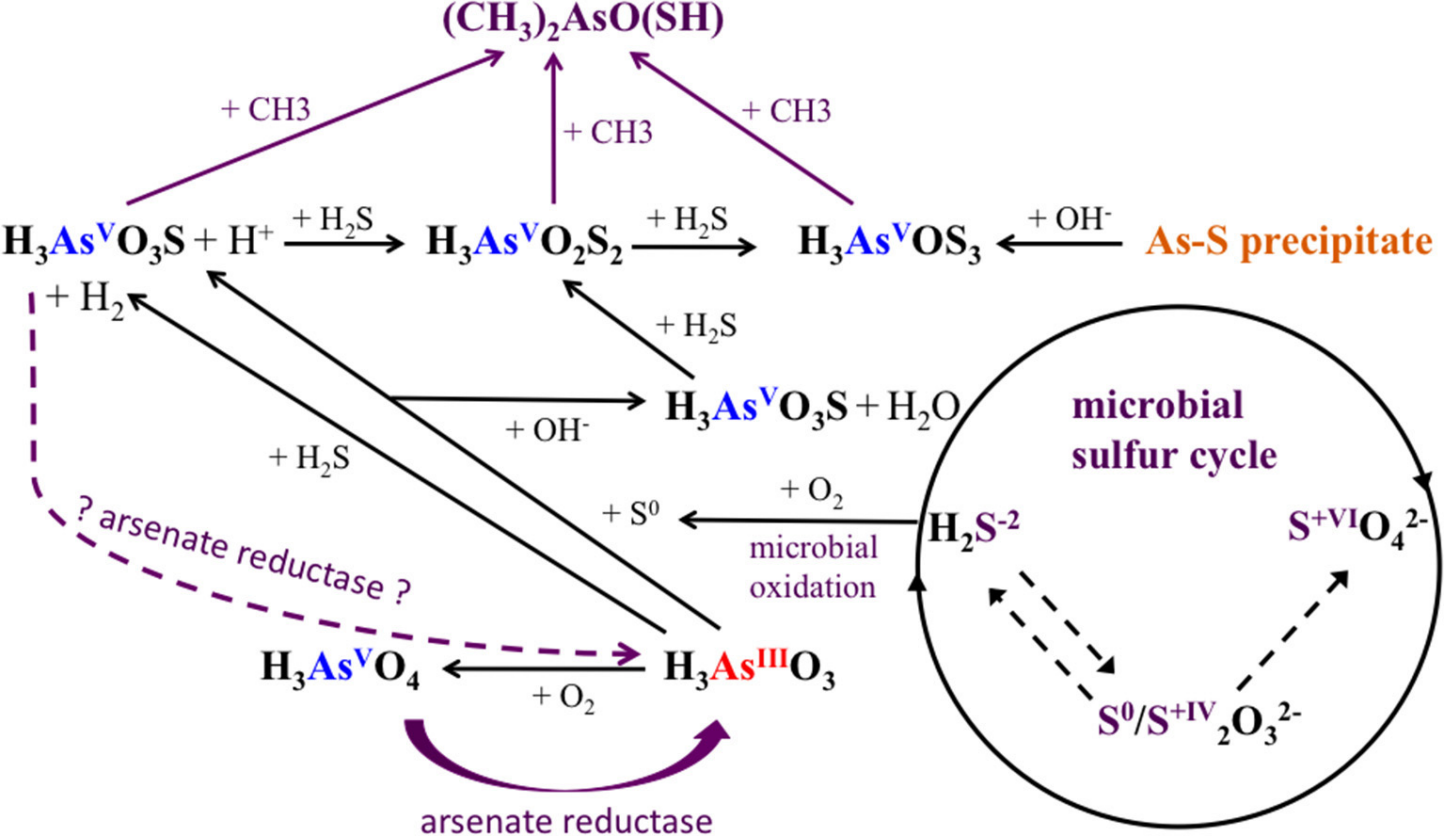
**Arsenic and sulfur cycle influenced by microorganisms in Champagne Pool**. H_3_AsO_3_, arsenite; H_3_AsO_4_, arsenate; H_3_AsO_3_S, monothioarsenate; H_3_AsO_2_S_2_, dithioarsenate; H_3_AsOS_3_, trithioarsenate; (CH_3_)_2_AsO(SH), dimethylmonothioarsenate. purple: biotic reactions.

Alongside the indirect impacts on arsenic transformation from microbial sulfur cycling, the metagenomic data for all sites revealed the presence of arsenic resistance genes (Figure 8). The dominance of the *ars* operon at all sites (Table 4) supports the high degree of utility and conservation of this arsenic resistance mechanism. The smallest functional *ars* operon, *arsRB*, detected at all sites (Table 4), was co-present with the arsenical pump-driving ATPase-encoding gene *arsA* (Table 4), which may enhance arsenite exclusion from cells. Some microbes at CPp and AP may also benefit from the arsenite resistance enhancer encoding gene *arsH* found in the metagenomes of these sites (Table 4). At AP, detection of the gene encoding for ArsD, which increases the affinity of ArsA to arsenite, would further enhance cellular arsenite exclusion. Although, statistical analysis did not show a significant change in the proportion of *arsB* to *acr3* from CPc to AP, a high proportion of the gene *acr3* at AP may reflect increasing microbial community diversity and the wider phylogenetic distribution of *acr3* compared to *arsB* (Achour et al., 2007; Fu et al., 2009). The detection of the arsenate reductase gene *arsC* throughout all sites (Table 4) may reflect the persistence of (thio)arsenate, as confirmed by arsenic speciation analysis (Figure 2). At CPp, CPr, and AP, *arsC* is accompanied by the respiratory arsenate reductase gene subunits of *arr* from which only the combination of *arrAB* at AP is functional (Table 4). In addition to arsenate, the ArsC and Arr enzymes likely also recognize thioarsenate, with arsenic in the same oxidation state as in arsenate (Figure 8). From these findings, we speculate an early origin for arsenate reductase, as thioarsenate levels would presumably be elevated in sulfidic hot springs on the early Earth.

The absence of the arsenite oxidase gene *aio* at all sites implies the *ars* operon as the sole arsenite resistance mechanism for coping with high arsenite levels at Champagne Pool (Figure 2), although there is a possibility that a newly identified arsenite oxidase encoding *arxA* gene (Zargar et al., 2010, 2012) has not been correctly annotated in MG-RAST. However, the implication of the *ars* operon as the sole arsenite resistance mechanism is an early prevalence of a purely detoxification mechanism over an arsenic resistance pathway coupled to energy conservation (*aio*). This hypothesis is perhaps supported by the detection of abundant sulfur and hydrogen oxidizing microorganisms (Figure 4), and sulfur and H_2_ respiratory genes, (Table 4) in the Champagne Pool metagenome for all sites. Either of these respiratory pathways would allow a cell to conserve more energy than could be obtained from arsenite oxidation.

The detection of the organic arsenic species dimethylmonothioarsenate in the outflow channel of Champagne Pool, CPc, revealed direct microbial control over arsenic speciation (Figure 8). The occurrence of methylated arsenic species can be uniquely attributed to arsenic-methylating microorganisms (Bentley and Chasteen, 2002). This resistance mechanism transforms arsenate and arsenite into mono-, di- and trimethylated arsenic, prior to excluding these species from the cell (Bentley and Chasteen, 2002). The trimethylated arsenic species arsine is highly volatile, and was therefore not included in our analyses. Interestingly, the occurrence of the organic arsenic species dimethylmonothioarsenate (Figure 2) corresponds in location (CPc) with a significant increase in sequences closely related to the family *Aquificaceae* (Figure 4). This family, with genera *Aquifex, Hydrogenobacter*, and *Hydrogenivirga*, all detected in CPc samples, is known for H_2_ oxidation. Molecular hydrogen is abundant at Champagne Pool, generated from hydrothermal reactions (Giggenbach et al., 1994), and is oxidized aerobically by microbes with O_2_ as the electron acceptor. The abundance of *Aquificaceae* at CPc may therefore be related to increased DO saturation, which reached a maximum at the outflow channel (Table 1). The family of *Aquificaceae* belongs to the order *Aquificales*, which is known to possess a functional arsenic methylation mechanism (Takacs-Vesbach et al., 2013). Typically, the gene *arsM* is associated with arsenic methylation (Qin et al., 2006), and may be co-expressed with the *ars* operon. However, no *arsM* sequences were detected at CPc. Nonetheless, the co-appearance of methylated arsenic and a significant increase in *Aquificaceae* supports the inference that As-methylation via an alternate mechanism to ArsM can likely be attributed to this group at CPc. These results reveal a potentially interesting role for methylation as an arsenic tolerance strategy in early geothermal springs.

In summary, quantitative arsenic and sulfur speciation of acid-sulfide hot spring waters provided the biogeochemical context in this study for understanding microbial community composition and functionality at Champagne Pool, Waiotapu, New Zealand. Analyses of community diversity; and arsenic resistance and sulfur cycling genes revealed several potential direct and indirect impacts of various microbial groups on coupled arsenic and sulfur cycling. Principally, the distributions of (thio)arsenate and the arsenate resistance genes *arsC* and *arr* suggest an ancient evolution for arsenate reductase that does not invoke arsenic oxidation by molecular oxygen. Further, our results support evolutionary prioritization of arsenite detoxification via the *ars* operon over oxidation via arsenite oxidase. Finally, the combination of abundant *Aquificaceae* relatives and the unique appearance of dimethylmonothioarsenate in the outflow channel of Champagne Pool suggests an important role for thermophilic arsenic methylation in the evolution of arsenic tolerance strategies.

### Conflict of interest statement

The authors declare that the research was conducted in the absence of any commercial or financial relationships that could be construed as a potential conflict of interest.

## Supplementary material

Information about the preparation of arsenic thioanion standards, measurement details for arsenic thioanions, a table with basic cation and anion data for Champagne Pool, HPLC-ICPMS chromatograms of arsenic species at the Champagne Pool sites, SEM images of the orange floc at the rim and outflow channel of Champagne Pool illustrating As-S precipitation and elemental sulfur and rarefaction curves illustrating the microbial community richness at the Champagne Pool sites are included in the supplementary material online.
