# Supplementary material for: Microbial contributions to coupled arsenic and sulfur cycling in the acid-sulfide hot spring Champagne Pool, New Zealand
Source: Front Microbiol. 2014 Nov 4;5:569. doi: 10.3389/fmicb.2014.00569 (PMC4220137; doi:10.3389/fmicb.2014.00569)
Supplement: Supplementary file 1 [file DataSheet1.DOCX]

***Supplementary Material***

**Microbial contributions to coupled arsenic and sulfur cycling in the acid-sulfide hot spring Champagne Pool, New Zealand**

**Katrin Hug^1*^, William A. Maher^2^, Matthew B. Stott^3^, Frank Krikowa^2^, Simon Foster^2^, John W. Moreau^1^***

^1^School of Earth Sciences, University of Melbourne, Melbourne, VIC, Australia

^2^Institute for Applied Ecology, University of Canberra, Canberra, ACT, Australia

^3^GNS Science, Wairakei, New Zealand

*** Correspondence:** Katrin Hug and John W. Moreau, Geomicrobiology Laboratory, School of Earth Sciences, University of Melbourne, Parkville VIC, 3010, Australia. [katrin.hug@gmail.com](mailto:jmoreau@unimelb.edu.au), [jmoreau@unimelb.edu.au](mailto:jmoreau@unimelb.edu.au)

1. **Supplementary Data**

## 1.1 Preparation of arsenic thioanion standards

Sodium monothioarsenate (Na_3_AsO_3_S*7H_2_O) was synthesized by adding 1.44 g sulfur to a mixture of 5 g As_2_O_3_ and 6 g NaOH in 20 ml H_2_O and heating to 100 °C for two hours. Excess sulfur was filtered off and the solution cooled slowly to 4 °C. The colorless crystals obtained were dried under vacuum for one hour. Sodium dithioarsenate (Na_3_AsO_2_S_2_*7H_2_O) was synthesized by adding 5.67 g sulfur to a mixture of 5 g As_2_O_3_ and 6 g NaOH in 20 ml H_2_O and heating to 70°C for two days. Excess sulfur was filtered off and the solution cooled slowly to 4 °C. The colorless rhombic crystals obtained were dried under vacuum for one hour and ready for use. All other sulfur-arsenic compounds were obtained by mixing various arsenic and sulfur concentrations (with sulfur:arsenic mass ratios of 1:1, 2:1, 3:1 and 4:1 mass/mass). All thioarsenate compounds were used for qualitative identification of arsenic species.

## 1.2 Arsenic thioanion measurement details

Mobile phase A was ultraclean H_2_O and mobile phase B 100 mM NaOH. The gradient was 1% B to 10% B in 5 min, 10% B to 100% B in 15 min, hold 100% B for 5 min, 1% B in 5 mins, for a total analysis time of 30 min. The eluent from the column was directed to a Dionex ASRS 300 (Sunnyvale, USA) (4mm) anion self-generating suppressor. The eluent flow rate was 1 ml min^-1^, the regenerant flow rate (ultrapure H_2_O) was 4 ml min^-1^ and the current was held constant at 100 mA. The eluent from the suppressor was directed to a Perkin Elmer NexionD ICP-MS through PEEK tubing (25 cm x 1.6 mm OD x 0.13 mm ID) to a Meinhard® glass micro concentric nebulizer with a glass cyclonic spray chamber. The ICP-MS was operated in standard mode and was equipped with nickel and an aluminum cone; plasma gas was 17 l min^-1^ and auxiliary gas 1.2 l min^-1^. Nebulizer gas flows were optimized daily and were between 0.82 – 0.84 ml min^-1^. RF power was 1250 W and a dwell time of 200 ms per element.

1. **Supplementary Figures and Tables**

## Supplementary Tables

**Supplementary Table 1. Basic cations and anions at Champagne Pool sites, Waiotapu, New Zealand.**

| Site ID    Cations (mg l^-1^) | CPp | CPr | CPc | AP |
| --- | --- | --- | --- | --- |
| Al | 0.24  ±0.01 | 0.206  ±0.01 | 0.212  ±0.008 | 0.161  ±0.001 |
| Mg | 0.061  ±0.002 | 0.05  ±0.001 | 0.054  ±0.004 | 0.054  ±0.002 |
| Na | 1177  ±1 | 1162  ±4 | 1180  ±2 | 1196  ±4 |
| Si | 491  ±2 | 484  ±2 | 495  ±2 | 497  ±1 |
| Fe | <0.08 | <0.08 | <0.08 | <0.08 |
| Anions (mg l^-1^) | **CPp** | **CPr** | **CPc** | **AP** |
| Cl^-^ | 1907  ±48 | No data | No data | No data |
| HCO3^-^ | 127  ±6 | No data | No data | No data |

## Supplementary Figures


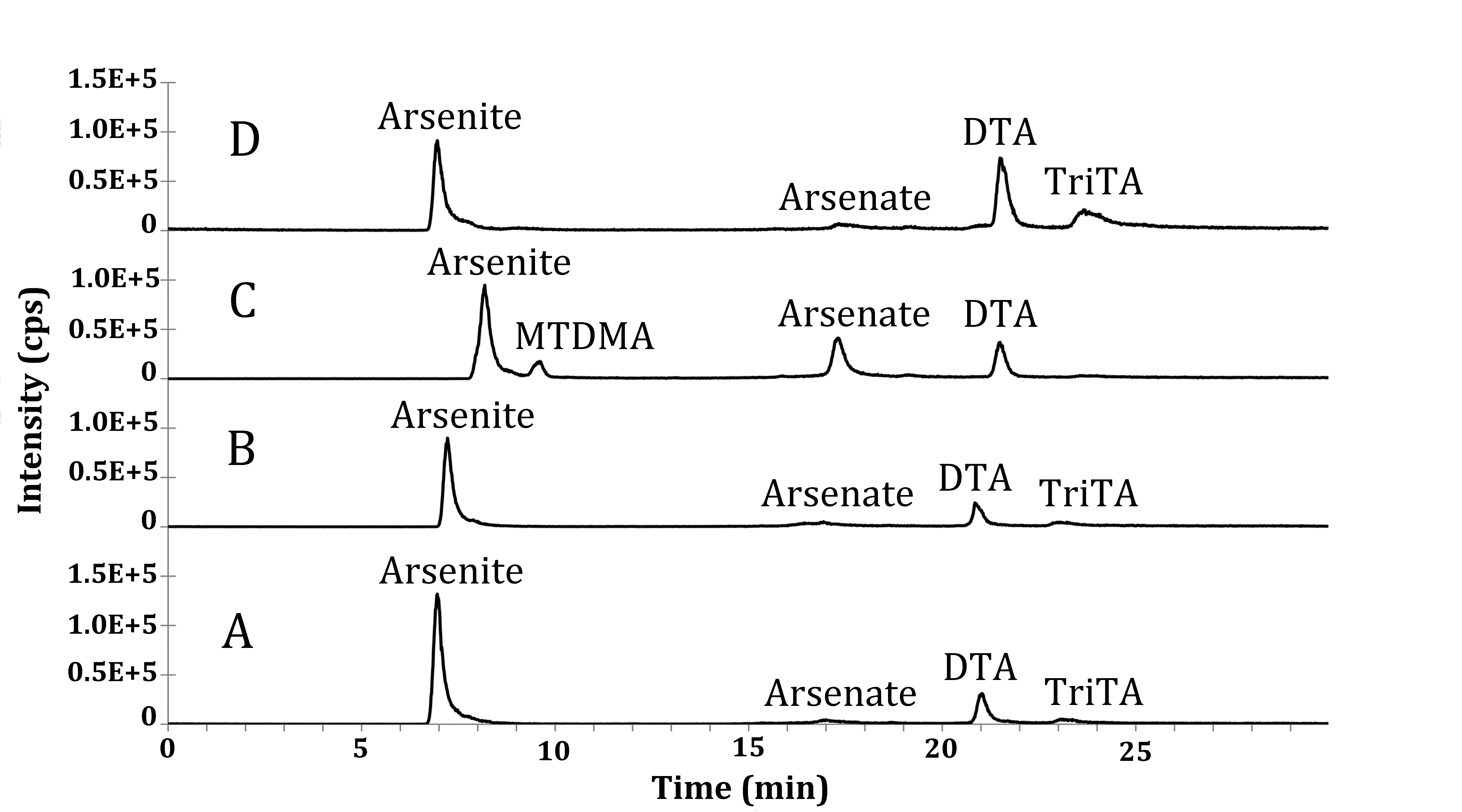


**Supplementary Figure 1.** **HPLC-ICPMS chromatograms of arsenic species at Champagne Pool sites.** (A) central pool (CPp); (B) rim of pool (CPr); (C) outflow channel (CPc); (D) “Artist’s Palette” terrace (AP). DTA: dithioarsenate, TriTA: trithioarsenate, MTDMA: dimethylmonothioarsenate. cps: counts per second.








**Supplementary Figure 2.** **SEM images of As-S precipitate (A) and elemental sulfur (B) at the rim (CPr) and outflow channel (CPp) of Champagne Pool.** y-axis: cps/eV= counts per seconds/electron volt, x-axis: keV= kiloelectron volt.


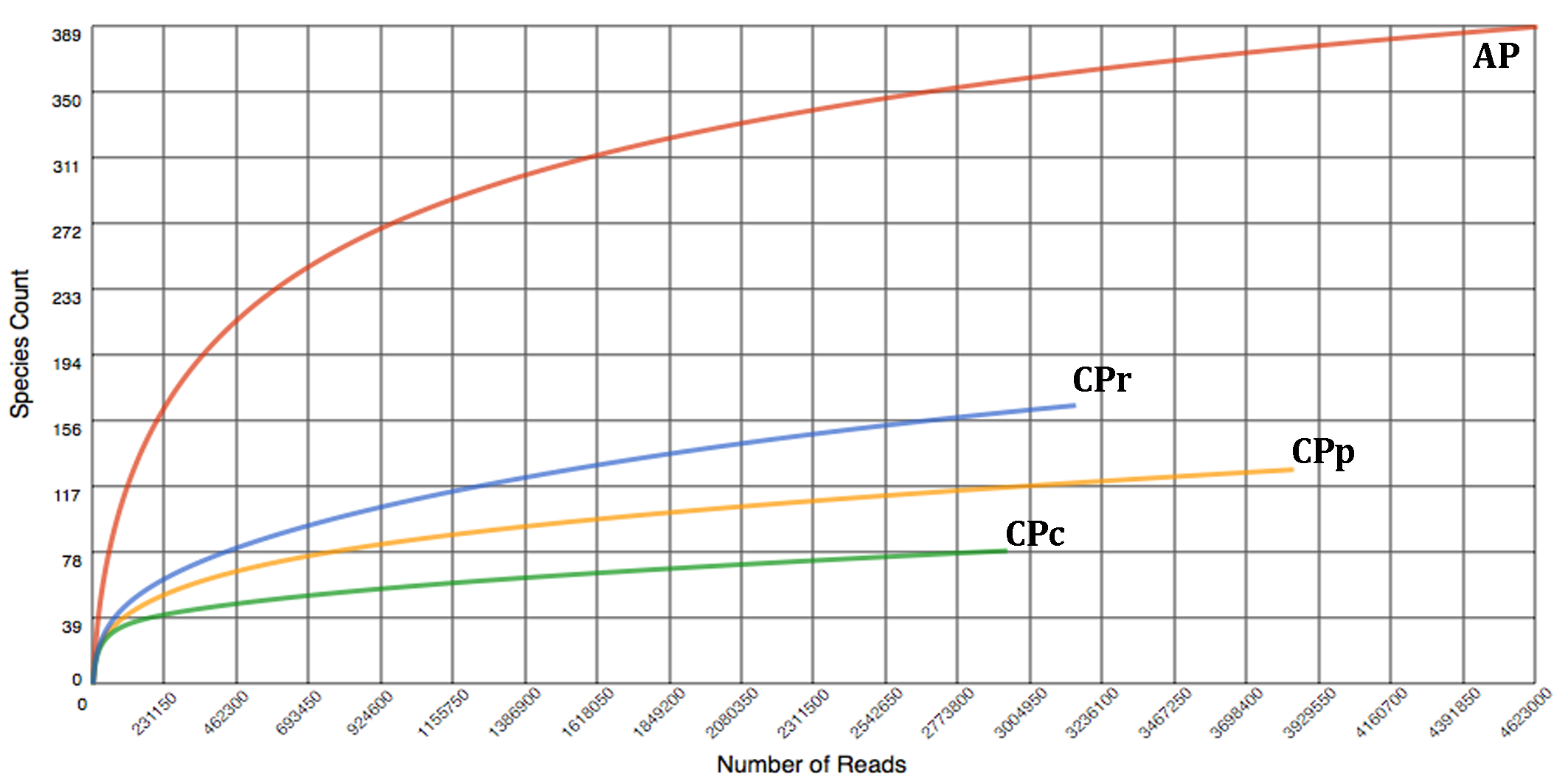


Supplementary Figure 3. Microbial species richness (16S rRNA gene) at Champagne Pool sites. The richness of the microbial community is illustrated as the species observed in the individual sample plotted against the number of reads in an individual sample. CPp: central pool, CPr: rim of pool, CPc: outflow channel, AP: “Artist’s Palette” terrace.
